# The burden of endometriosis on quality of life in Danish women: an analysis of the Danish Blood Donor Study

**DOI:** 10.1186/s12916-025-04398-z

**Published:** 2025-10-14

**Authors:** Lisette J. A. Kogelman, Dorte Rytter, Lone Hummelshoj, Karina Ejgaard Hansen, Ulrik Bak Kirk, Juliane Lyng Beauchamp, Jakob Thaning Bay, Mie Topholm Bruun, Nanna Brøns, Christian Erikstrup, Bitten Aagaard, Bertram Dalskov Kjerulff, Christina Mikkelsen, Susan Mikkelsen, Sisse Rye Ostrowski, Ole Birger Pedersen, Erik Sørensen, Henrik Ullum, DBDS Genomic Consortium, Mie Topholm Bruun, Nanna Brøns, Christian Erikstrup, Christina Mikkelsen, Susan Mikkelsen, Sisse Rye Ostrowski, Ole Birger Pedersen, Erik Sørensen, Henrik Ullum, Jakob Bay, Andrea Barghetti, Mette Skou Bendtsen, Jens Kjærgaard Boldsen, Søren Brunak, Alfonso Buil Demur, Johan Skov Bundgaard, Lea Arregui Nordahl Christoffersen, Maria Didriksen, Khoa Manh Dinh, Joseph Dowsett, Josephine Gladov, Daniel Gudbjartsson, Thomas Folkmann Hansen, Dorte Helenius Mikkelsen, Lotte Hindhede, Henrik Hjalgrim, Jakob Hjorth von Stemann, Bitten Aagaard Jensen, Kathrine Kaspersen, Bertram Dalskov Kjerulff, Lisette J. A. Kogelman, Mette Kongstad, Line Hjorth Sjernholm Nielsen, Janna Nissen, Frederikke Byron Pedersen, Liam James Elgaard Quinn, Þórunn Rafnar, Klaus Rostgaard, Andrew Joseph Schork, Michael Schwinn, Hreinn Stefánsson, Jacob Træholt, Unnur Þorsteinsdóttir, Thomas Werge, Kari Stefansson, Palle Duun Rohde, Mette Nyegaard, Anne Karmisholt Grosen, Christian Lodberg Hvas, Valgerdur Steinthorsdottir, Kari Stefansson, Karina Banasik, Palle Duun Rohde, Henriette Svarre Nielsen, Mette Nyegaard

**Affiliations:** 1 Genomic Medicine, Department of Health Science and Technology, Aalborg University, Aalborg, Denmark; 2 Translational Research Centre, Copenhagen University Hospital, Glostrup, Denmark; 3 Danish Headache Center, Department of Neurology, Copenhagen University Hospital, Glostrup, Denmark; 4 Department of Public Health, Aarhus University, Aarhus, Denmark; 5 Endometriosis.org, London, UK; 6 Research Unit for General Practice, Aarhus, Denmark; 7 Department of Obstetrics and Gynaecology, Copenhagen University Hospital, Hvidovre, Denmark; 8 Department of Clinical Immunology, Zealand University Hospital, Køge, Denmark; 9 Department of Clinical Immunology, Odense University Hospital, Odense, Denmark; 10 Department of Clinical Immunology, Rigshospitalet, Copenhagen University Hospital, Copenhagen, Denmark; 11 Department of Clinical Immunology, Aarhus University Hospital, Aarhus, Denmark; 12 Department of Clinical Medicine, Aarhus University, Aarhus, Denmark; 13 Department of Clinical Immunology, Aalborg University Hospital, Aalborg, Denmark; 14 Novo Nordisk Foundation Center for Basic Metabolic Research, Faculty of Health and Medical Science, University of Copenhagen, Copenhagen, Denmark; 15 Department of Clinical Medicine, Faculty of Health and Medical Sciences, University of Copenhagen, Copenhagen, Denmark; 16 Statens Serum Institut, Copenhagen, Denmark; 17 Department of Clinical Microbiology, Aarhus University Hospital, Aarhus, Denmark; 18 Department of Hepatology and Gastroenterology, Aarhus University Hospital, DK-8200 Aarhus N, Denmark; 19 Department of Clinical Medicine, Aarhus University, DK-8200 Aarhus N, Denmark; 20 deCODE Genetics-Amgen, Reykjavik, Iceland; 21 Section for Bioinformatics, DTU Health Tech, Technical University of Denmark, Kongens Lyngby, Denmark

**Keywords:** Endometriosis, Comorbidities, Polygenic score, Genetic burden

## Abstract

**Background:**

Endometriosis is a complex condition with a wide range of comorbidities. It is widely underdiagnosed, with a diagnostic delay of 4 to 10 years, potentially leading to worsened disease progression and a higher burden of comorbidities affecting quality of life. Understanding the link between endometriosis and its comorbidities is essential for improving early detection of the disease.

**Methods:**

We analysed data from 953 women with a clinical diagnosis of endometriosis and 23,652 age-matched female controls enrolled in the Danish Blood Donor Study, using a case-control design. Participants completed one to four questionnaires covering a wide range of potential comorbidities; genetic data were available for a subset of participants. First, we compared the potential comorbidities between women with endometriosis and controls. Next, we investigated whether a polygenic score (PGS) for endometriosis was associated with those comorbidities. Lastly, we investigated whether women with a high genetic burden of endometriosis (highest PGS decile) experienced similar comorbidities to those diagnosed with endometriosis.

**Results:**

Women with endometriosis experienced challenges in conception, gastrointestinal symptoms, and disturbed sleep patterns, compared to age-matched controls. The endometriosis PGS showed to be a predictor for endometriosis (OR per unit PGS = 1.43, 95% CI = 1.32–1.55). Gastrointestinal symptoms were also nominally associated with the endometriosis PGS, suggesting shared genetic pathways. Women without a diagnosis of endometriosis but with a high genetic burden of endometriosis did not suffer from the same wide range of comorbidities as women diagnosed with endometriosis.

**Conclusions:**

Our findings highlight the complex genetic and clinical relationships between endometriosis and its comorbidities, emphasizing the need for future research investigating potential endometriosis subtypes.

**Supplementary Information:**

The online version contains supplementary material available at 10.1186/s12916-025-04398-z.

## Background

Endometriosis is a complex condition that imposes a substantial economic burden [1, 2]. It is estimated to affect approximately 10% of reproductive-age women worldwide, although prevalence varies depending on diagnostic criteria and population characteristics [3, 4]. Endometriosis extends far beyond its primary manifestation of endometrial-like tissue outside the uterus to a large range of comorbidities, such as infertility, persistent abdominal pain, gastrointestinal complications, migraine, and challenges related to mental health [5–10]. Endometriosis is widely underdiagnosed [11–13], with a diagnostic delay of 4 to 10 years [14–17] which leads to delayed treatment and prolonged patient suffering [9, 18]. This diagnostic delay not only exacerbates disease progression but may also contribute to an increased burden of comorbid conditions. Understanding the relationship between endometriosis and its comorbidities is crucial for enabling early detection, improving patient outcomes, and building comprehensive management strategies.

Although the exact aetiology of endometriosis is still not fully understood, substantial evidence suggests a significant genetic component in the risk of developing the condition, with an estimated heritability of around 50% [19, 20]. Genome-wide association studies (GWAS) have identified 42 common genetic loci associated with the risk of endometriosis [21] with moderate to small effect sizes. Due to the moderate to small effect sizes, common in complex diseases [22], there is considerable interest in consolidating the impacts of multiple genetic risk variants into a combined score. A commonly employed scoring method involves calculating the sum of risk alleles, with each single nucleotide variant weighted by its GWAS effect size—known as a polygenic score (PGS) [23, 24]. Because the PGS has a theoretical relationship with the genetic liability model of polygenic diseases, it has led to widespread use of PGS in biomedical research [24]. We hypothesize that the observed comorbidities are intricately linked to the genetic burden of endometriosis.

In this study, we investigate 37 comorbidities of endometriosis in up to 24,605 individuals participating in the Danish Blood Donor Study (DBDS) [25]. By constructing a genome-wide PGS for endometriosis, we assess to what degree the genetic burden of endometriosis was associated with comorbidities of endometriosis. This approach enhances our understanding of the impact of endometriosis on quality of life and the potential association of endometriosis with genetic factors.

## Methods

### Study population

From March 2010 to December 2022, voluntary blood donors were recruited as part of the Danish Blood Donor Study (DBDS), an ongoing prospective population-based research cohort [25]. Eligible participants were aged 18–67 years and weighing > 50 kg. Blood donors are subject to strict eligibility criteria as defined by the Transfusion Medicine Standards (https://dski.dk/gaeldende-version/). Participating blood donors completed between one and four different questionnaires during the recruitment period. The first version was a paper-based questionnaire, whilst the following three were digital questionnaires. Questionnaires covered questions on self-experienced physical and mental health (including the Short Form Health Survey 12 [SF-12] [26]), smoking habits, sleep patterns, allergies, attention-deficit/hyperactivity disorder (ADHD), migraine, depression, pregnancy, gastrointestinal symptoms, and many other health-related conditions.

### Diagnosis of endometriosis

The Danish National Patient Registry (DNPR) contains data on all in- and outpatients discharged from Danish hospitals since 1977 and, since 2002, it also includes records from Danish private hospitals. An endometriosis diagnosis was defined based on the presence of any of the codes: ICD-8 codes 62.530 and 62.532–62.539 (< 1994) and ICD-10 codes N80.1–N80.9 (≥ 1994) in the DNPR, regardless of surgical or histologic verification. Diagnoses of adenomyosis (ICD8: 62,531, ICD10: N80.0) were not included; individuals with an adenomyosis diagnosis only, and no other endometriosis-related disease codes, were not considered as cases. The date of diagnosis was based on the hospital admission date for the first endometriosis diagnosis. For each questionnaire, a participant was counted as an endometriosis case when, at the time of filling out the questionnaire, the individual had a diagnosis of endometriosis in DNPR. Diagnoses were subdivided based on their ICD codes into ‘deep endometriosis’ (ICD8: 62,535, 62,536; ICD10: N80.4, N80.5), ‘ovarian endometriosis’ (ICD8: 62,530; ICD10: N80.1), ‘peritoneal endometriosis’ (ICD8: 62,532, 62,533; ICD10: N80.2, N80.3), and ‘other endometriosis’ (ICD8: 62,534, 62,537, 62,538, 62,539; ICD10: N80.6, N80.8, N80.9). Severity of endometriosis was divided into severe (i.e. stage III/IV endometriosis including ‘deep’ and/or ‘ovarian’- endometriosis) and moderate (i.e. stage I/II endometriosis including ‘peritoneal’ and/or ‘other’- endometriosis) according to the r-ASRM score [27]. As this classification does not always align perfectly with anatomical subtypes, this categorization should be considered a proxy rather than a direct translation.

### Age-matching

Female blood donors with an endometriosis diagnosis were significantly older than females without endometriosis (45.7 *versus* 44.6 years, *P* = 2.28 × 10^−3^). Therefore, for each questionnaire, an age-matched female study population was selected from the DBDS cohort, with a case:control ratio of ~ 1:25 (Table 1). At the time of filling in the respective questionnaires, the ‘case’ had to have an endometriosis diagnosis; ‘controls’ were not diagnosed with endometriosis at any point throughout the complete study period (8 March 1977–9 November 2022) and all had genetic data available.

**Table 1:** Overview of questionnaires and a breakdown of the number of individuals (*n*) with questionnaire (*Q*) and genetic (*G*) data available

Questionnaire	Time	*n*	
		Endometriosis (*Q*|*G*)	Control
**DBDS1**	03/2010–01/2015	526|471	11,572
**DBDS2**	05/2015–06/2018	350|266	10,016
**DBDS3**	06/2018–03/2020	308|190	7051
**DBDS4**	11/2020–11/2022	301|153	6575
**Total (unique)**	03/2010–11/2022	953|649	23,652

*n* count, *Q* questionnaire data available, *G* genetic information available. Selected controls all had both questionnaire and genetic data available

### Genetic data

The majority of the DBDS participants have genetic information available (*n* = 100,146), which established the DBDS Genomic Cohort [28]. A total of 649 women with endometriosis and all 23,652 age-matched female controls had genetic information available (Table 1). At inclusion, a blood sample for DNA analyses was collected and saved in the biobank. DNA genotyping of the DBDS samples was obtained at deCODE Genetics using the Global Screening Array by Illumina. The raw genotype data was processed at deCODE Genetics simultaneously for genotype calling, quality control and genotype imputation using an in-house reference panel consisting of the UK 1000 Genomes Project phase 3, HapMap reference and an in-house dataset of > 6000 Danish whole genome sequences [29]. QC included removal of duplicates, sex mismatches, samples with > 5% missingness, variants with > 10% missingness, and variants with Hardy–Weinberg equilibrium *P* < 1 × 10⁻⁵. Participants were included based on genetic principal component (PC) analysis, selecting individuals within 5 standard deviations of those with Danish-born parents. PCs were derived from a linkage disequilibrium (LD)-pruned set of 30,966 genetic variants using PLINK [30], and calculated with flashPCA [31]. The resulting PCs were used in downstream PGS analyses to adjust for population stratification. Identification of relatedness was done using PLINK2 based on the king-cutoff (value < 0.0884) command. Any related individuals were removed from the data (up to second degree relatives) to avoid inflated predictions in subsequent analysis.

### Assessing comorbidities

All questions used in this study are presented in Supplementary File 1: Table S1; participants only received the Danish question. Questionnaire data was pre-processed at question level by excluding individuals who answered, ‘Do not know’. Several questions were part of a scoring system, where multiple questions were combined into a single scale. Insomnia was defined as experiencing at least one of three symptoms ≥ 3 times per week: (1) difficulty falling asleep within 30 min, (2) waking up too early and unable to fall back asleep, or (3) woken up during the night or early morning. Daytime fatigue was defined as experiencing at least one of three symptoms ≥ 3 times per week: (1) extreme tiredness during the day, (2) an irresistible urge to sleep at work/school, and (3) an irresistible urge to sleep during spare time. Restlessness in legs during sleep was identified when participants reported experiencing restless legs ≥ 3 times per week. Physical and mental health status was measured using the 12-item Short Form Health Survey (SF-12), which yields two composite scores: the physical component summary score (PCS) and the mental component summary score (MCS) [32]. Scores were calculated using weighted item endorsements, yielding values between 0 and 100, with higher scores reflecting better quality of life [33]. Perceived stress was measured using the 10-item Cohen’s Perceived Stress Scale (PSS) [34]. The questions were answered on a 5-point Likert scale, asking the respondents to indicate how often they experience a specific stress symptom ranging from ‘0 = never’ to ‘4 = very often’, resulting in a score ranging from 0 to 40. Depression was measured using the Major Depression Inventory (MDI): a validated self-report questionnaire of 10 items ranging from 0 to 5 [35, 36]. The MDI score was calculated as the sum of all 10 items, including only the item with the highest score out of item 8a and 8b, and similarly the one with the highest score out of item 10a and 10b. Depression was analysed both on a continuous scale (MDI score) and classification of depression using a cut-off of > 20.

As participants might have donated blood more than once during the 12-year period, they may have participated in multiple questionnaires. When a question was asked more than once (e.g. smoking behaviour and body mass index), the answer in the most recent questionnaire was used in this study. For women with endometriosis, the most recent questionnaire after having received an endometriosis diagnosis was used.

Besides the questionnaires, the Danish Medical Birth Register was used to investigate the age of first birth [37].

### Polygenic score for endometriosis

The endometriosis PGS, reflecting genetic susceptibility to the disease, was constructed using GWAS summary data from the most recent endometriosis meta-GWAS [21]. To avoid sample overlap and ensure ancestry homogeneity, we used GWAS summary statistics that excluded DBDS participants, the 23andMe cohort (due to lack of publicly available data), and the Japanese cohort (to restrict analyses to individuals of European ancestry). This resulted in a GWAS summary dataset comprising 23,112 cases and 429,677 controls, from European ancestry. The endometriosis PGS was calculated using LDpred2 software [38] using the auto option with default settings. The endometriosis PGS was standardized to a mean of zero and standard deviation of 1, and when used to measure the prediction accuracy for the comorbidities, the endometriosis PGS was rescaled to a mean of zero and one unit standard deviation corresponding to a twofold genetic increased risk for endometriosis in the target population; this was done by subtracting the mean PGS from each individual’s PGS and then multiplying by log(OR)/log(2), where the odds ratio (OR) was obtained from the model predicting endometriosis (see below).

### Statistical analyses

Differences in comorbidities between those diagnosed with endometriosis and female participants without were compared using Student’s *t*-tests (continuous traits), ${\chi }^{2}$-squared test (binomial traits) or Wilcoxon signed-rank test (ordinal traits). To formally test for differences while adjusting for potential confounders, we estimated *p*-values using generalized linear models (GLMs) adjusted for age and blood donation region. Adjustment for region was included due to observed regional differences in endometriosis diagnosis, consistent with a previous study [39]. Resulting *P*-values were corrected for multiple testing, based on the number of tests performed across the whole study (*n* = 38) using the false discovery rate (FDR) [40]. Statistical differences between endometriosis cases and controls were determined significant when *P*_FDR_ < 0.05. Further, we investigated whether there were differences in comorbidities between women with stage I/II endometriosis compared to women with stage III/IV endometriosis using the same model as described above. Similar, resulting *p*-values were corrected for multiple testing using the FDR, and determined significant when *P*_FDR_ < 0.05.

Only comorbidities that were significantly associated with endometriosis were subsequently associated with the endometriosis PGS using a logistic regression including age, region and the first five genetic PCs as covariates. The discriminative ability of the PGS was determined using the area under the receiver operating curve (AUC). Resulting *p*-values were corrected for multiple testing, based on the number of tests performed (i.e. those significantly associated with endometriosis on phenotype level; *n* = 11) using the FDR. Comorbidities that displayed an association with endometriosis PGS of *P*_FDR_ < 0.05 were considered significant. Significant predictions were presented as odds ratios with 95% confidence intervals, using the rescaled PGS, i.e. one standard deviation of the PGS corresponds to a twofold genetic increased risk for endometriosis.

To investigate whether individuals with a high genetic burden for endometriosis, but no endometriosis diagnosis, exhibit similar comorbidity profiles as women with diagnosed endometriosis, we identified a subset of controls whose PGS fell within the top decile of the PGS distribution. These high-PGS controls were compared to the remaining controls (i.e. not in the top decile) and to cases (i.e. women with diagnosed endometriosis), with the three groups being mutually exclusive. Only comorbidities that were significantly associated with endometriosis were investigated using logistic regression, adjusting for age, region, and the first five genetic principal components.

All statistical analyses were performed in R (v4.0.0).

## Results

**Fig. 1:**
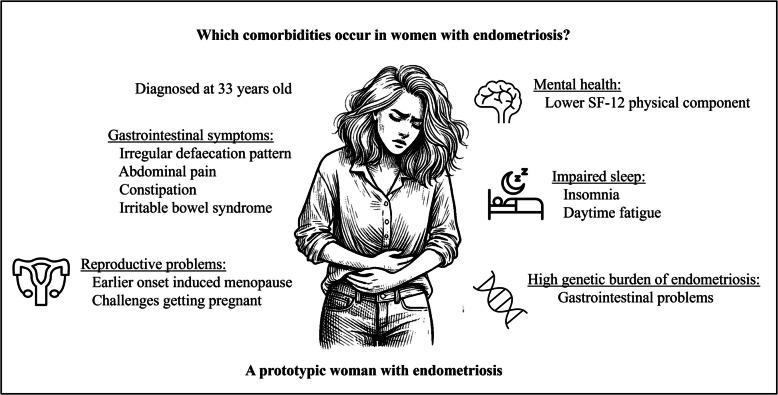
Summary of findings of comparing women with endometriosis with age-matched controls

From 24,605 females selected from the DBDS cohort for this study, 953 females were diagnosed with endometriosis at the time of filling in (one of) the questionnaires. A summary of our findings is presented in Fig. 1. Almost half of the women with endometriosis were diagnosed with one subtype of endometriosis, of whom 19 (2%) had deep infiltrating endometriosis, 140 (15%) had ovarian endometriosis, 100 (10%) had peritoneal endometriosis, and 354 (37%) had ‘other endometriosis’. Among the women diagnosed with two or more subtypes of endometriosis, the most common co-occurrences were ovary and other endometriosis (*n* = 172, 18%) and peritoneal and other endometriosis (*n* = 139, 15%). Of the 953, 534 (56%) were categorized as moderate endometriosis (stage I/II) and 419 (44%) as severe endometriosis (stage III/IV).

### General characteristics of women with endometriosis

On average, endometriosis was diagnosed at the age of 33.0 (SD = 8.3) years. Before age-matching, we found a prevalence proportion for endometriosis of 1.28%. Even though the prevalence proportions differed by region of Denmark (*P* < 0.001), we did not see a significant difference in age at diagnosis between the regions (*P* = 0.05). Women diagnosed with endometriosis had similar body mass index (BMI), standing height, and waist circumference as the female controls (Table 2), and there was no difference in the frequency of smoking (or smoking in the childhood home) observed between women with endometriosis and their age-matched controls (Table 2).

**Table 2:** Descriptive statistics of the cohort

Characteristics	*n* (*E*/*C*)	Endometriosis	Controls	*P*_FDR_
BMI, mean (SD)	941/23,487	25.9 (4.8)	25.6 (4.7)	1.00
Height, mean (SD)	951/23,606	169.1 (6.2)	168.8 (6.0)	1.00
Waist circumference, mean (SD)	482/10,526	87.3 (11.1)	86.0 (11.4)	1.00
Smoking, %	950/18,517	15.7	15.4	1.00
Smoking in childhood home, %	524/11,357	74.6	65.5	1.00

Abbreviations: *n*, number of participants answered the question; *E*, women with endometriosis; *C*, controls; *P*_*PFDR*_, false discovery rate

### Genetic risk of endometriosis predicts endometriosis and its severity

**Fig. 2:**
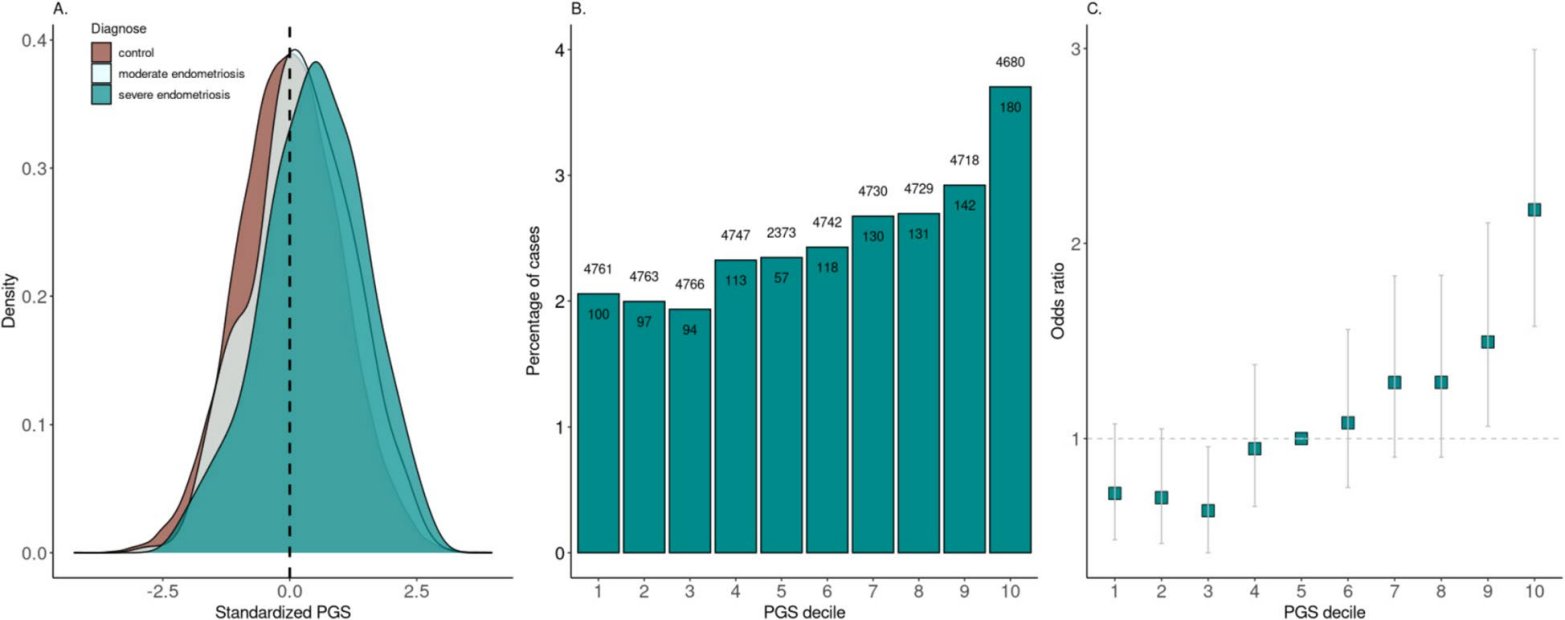
**A** Density plot of the endometriosis polygenic score (PGS) in healthy females, patients with moderate endometriosis, and patients with severe endometriosis; **B** PGS divided into deciles and the proportion of cases are counted per decile (numbers of controls are written above the bars, number of cases written in the bars); and **C** for each PGS decile, the odds ratio (OR) for endometriosis is estimated (error bars indicate standard error of the estimate; reference decile was set to decile 5)

The DBDS genomic cohort included 649 endometriosis cases and 23,652 age-matched controls. The average standardized endometriosis PGS was significantly higher in women with endometriosis than in controls (Fig. 2A), with an OR for endometriosis of 1.43 (95% CI = 1.32–1.55) and AUC of 0.66. The age of endometriosis diagnosis was not significantly associated with the endometriosis PGS (*P* = 0.99). The endometriosis PGS was higher in all endometriosis subtypes, except for ‘other’ (*P* = 0.55), compared to the controls (Table 3) and was higher among women with stage III/IV endometriosis than women with stage I/II endometriosis (*P* = 2.11 × 10^−3^). The endometriosis PGS was also higher in women with multiple endometriosis ICD codes compared to women with one endometriosis ICD code (OR = 1.30, 95% CI = 1.14–1.49, *P* = 3.79 × 10^−4^). Furthermore, individuals in the top 10 percent of PGS had more than double the risk for endometriosis compared to individuals within the 5th decile of PGS (Fig. 2B–C).

**Table 3:** Prediction of endometriosis at different locations using the polygenic score (PGS) of endometrioses

	n^*^	**PGS** (mean (SD))	**AUC**	***P***_**FDR**_
Endometriosis (any)	649	0.35 (1.01)	0.66	< 2.00 × 10^−16^
Deep	78	0.51 (1.07)	0.75	6.12 × 10^−6^
Ovarian	253	0.49 (0.99)	0.68	4.74 × 10^−15^
Peritoneal	215	0.47 (0.98)	0.69	7.34 × 10^−12^
Other	396	0.36 (1.03)	0.67	6.75 × 10^−13^

Abbreviations: *n*, number of cases; *PGS*, standardized polygenic score; *AUC*, area under the receiver operating characteristic curve; *P*_*FDR*_, false discovery rate

^*^Tested against all (*n* = 23,652) controls

### Reproductive health in women with endometriosis

The reproductive health of women with endometriosis was different to the age-matched female controls (Table 4). Women with endometriosis reported no earlier age at menarche compared to controls, neither did we find a difference in menstrual duration. Furthermore, among women who entered menopause at the time of filling out the questionnaire, we found an earlier age of induced onset of menopause of women with endometriosis (3 years earlier), but not in natural menopause, compared to controls. The severity of endometriosis was not associated with the age of menarche, menstrual duration, or onset of menopause (all *P*_FDR_ > 0.1, Supplementary File 1: Table S2). We found a higher prevalence of women with endometriosis who reported difficulties conceiving compared to controls (OR = 3.09, 95% CI = 2.45–3.91), though the proportion of women that had been pregnant (~ 60%) and age of first birth (~ 26 years) was similar. Among those who reported difficulties conceiving, the mean age at first pregnancy was also not significantly different between women with endometriosis (26.9 years) and controls (27.5 years; *P* = 0.34). Among women with endometriosis, there was no association between the severity of endometriosis and difficulties conceiving (*P*_FDR_ > 0.1, Supplementary File 1: Table S2). During pregnancy, there was no difference in nausea between women with endometriosis and controls.

**Table 4:** Reproductive characteristics in women with endometriosis versus controls

Characteristics	*n* (*E*/*C*)	Endometriosis	Controls	*P*_FDR_
Age of menarche, mean (SD)	512/11,226	13.0 (1.5)	13.2 (1.4)	0.10
Menstrual duration, mean (SD)	231/7048	5.0 (1.7)	5.0 (1.5)	1.00
Age of onset natural menopause, mean (SD)	80/2313	48.0 (5.7)	49.7 (4.2)	0.27
Age of onset induced menopause, mean (SD)	150/956	40.0 (7.6)	43.0 (6.7)	5.59 × 10^−4^
Tried to conceive ≥ 6 months w/o success, %	306/6959	45.4	21.4	7.60 × 10^−15^
Have been pregnant, %	308/7027	58.1	60.7	1.00
Age first pregnancy, mean (SD)	72/1715	25.3 (3.8)	26.0 (3.8)	1.00
Nausea during pregnancy, %	175/4200	60.6	61.3	1.00

Abbreviations: *n*, number of participants answered the question; *E*, women with endometriosis; *C*, controls; *P*_*FDR*_, false discovery rate

The genetic risk of endometriosis was not associated with the onset of induced menopause (*P*_FDR_ = 1.00). No significant linear association was found between the endometriosis PGS and problems getting pregnant (*P*_FDR_ = 0.57).

### Gastrointestinal symptoms and bowel habits associated with endometriosis

We found that women with endometriosis more often reported an irregular defaecation pattern regularity (OR = 1.67, 95% CI = 1.23–2.25; Table 5). Moreover, women with endometriosis had more gastrointestinal symptoms, such as abdominal pain, postprandial pain, defaecation pain, and constipation, than the age-matched female controls (*P*_FDR_ < 0.05). Those symptoms may be in relation to the higher prevalence of self-reported irritable bowel syndrome among women with endometriosis (OR = 1.95, 95% CI = 1.41–2.69). Neither the gastrointestinal pain symptoms and defaecation pattern regularity, nor irritable bowel syndrome, were reported more often in cases of stage III/IV endometriosis compared to stage I/II endometriosis (*P*_FDR_ > 0.1, Supplementary File 1: Table S2). However, constipation occurred more often in women with stage III/IV endometriosis compared to women with stage I/II endometriosis (87.1% versus 71.1%, *P*_FDR_ = 0.03). We did not find a significant association between severity of endometriosis and self-reported coeliac disease, neither did we find an association with self-reported lactose intolerance.

**Table 5:** Gastrointestinal characteristics in women with endometriosis versus controls

Characteristics		*n* (*E*/*C*)	Endometriosis	Controls	*P*_FDR_
Defaecation pattern regularity, irregular, %			18.6	12.2	4.92 × 10^−3^
Gastrointestinal symptoms					
* Bloating*, %		292/6404	50.3	43.2	0.06
* Abdominal rumbling*, %		293/6398	42.7	38.8	0.44
* Acid regurgitation*, %		293/6431	14.0	10.8	0.31
* Heartburn*, %		295/6442	12.5	11.6	1.00
* Lack of appetite*, %		294/6420	6.5	6.3	1.00
* Nausea*, %		295/6443	14.2	11.5	0.26
* Vomiting*, %		293/6423	1.0	1.3	1.00
* Abdominal pain*, %		296/6497	29.7	18.3	1.63 × 10^−5^
* Pain during food intake*, %		296/6507	2.4	1.0	0.11
* Postprandial pain*, %		295/6494	14.9	8.0	2.66 × 10^−4^
* Defaecation pain*, %		295/6502	15.9	8.4	1.16 × 10^−4^
* Diarrhoea*, %		294/6483	11.2	10.8	1.00
* Constipation*, %		290/6417	20.3	12.5	3.60 × 10^−4^
Irritable bowel syndrome, %		250/5842	20.0	11.4	4.90 × 10^−4^
Lactose intolerance, %		258/5981	5.8	3.3	0.11
Gluten intolerance (celiac disease), %		260/6010	1.5	0.7	0.41

Abbreviations: *n*, number of participants answered the question; *E*, women with endometriosis; *C*, controls; *P*_*FDR*_, false discovery rate

The endometriosis PGS was not associated with defaecation pattern regularity (*P*_FDR_ = 1.00), though it did associate (nominally significant) with some of the gastrointestinal symptoms, i.e. abdominal pain (OR = 1.22, 95% CI = 1.01–1.47, *p* = 0.04, *P*_FDR_ = 0.55) and pain during food intake (OR = 2.60, 95% CI = 1.28–5.29, *p* = 0.01, *P*_FDR_ = 0.09). Furthermore, a doubling in genetic risk of endometriosis was associated with a 1.34 times greater risk of self-reported irritable bowel syndrome (95% CI = 1.06–1.69, *p* = 0.02, *P*_FDR_ = 0.17).

### Impaired sleep quality in women with endometriosis

We found that women with endometriosis suffered more often from insomnia (OR = 1.38, 95% CI = 1.13–1.67) and daytime fatigue (OR = 2.10, 95% CI = 1.53–2.88), but we found no difference in restless legs during sleep compared to controls (Table 6). We did not find an increase in sleep problems related to the severity of endometriosis (*P*_FDR_ > 0.1, Supplementary File 1: Table S2). Sleep characteristics, such as insomnia, daytime fatigue, and restless legs during sleep, were not associated with the endometriosis PGS (*P*_FDR_ = 1.00).

**Table 6:** Sleep characteristics in women with endometriosis versus controls

Characteristics	*n* (*E*/*C*)	Endometriosis	Controls	*P*
Insomnia, %	480/7505	36.7	28.7	0.03
Daytime fatigue, %	480/7464	10.4	5.4	1.43 × 10^−4^
Restless legs during sleep, %	183/3986	2.7	1.8	1.00

Abbreviations: *n*, number of participants answered the question; *E*, women with endometriosis; *C*, controls; *P*_*FDR*_, false discovery rate

### The mental health of those with endometriosis is not affected

The impact of health on an individual’s daily life was measured by the SF-12. We did not find any difference between women with endometriosis and their controls on the mental component of SF-12, though we found a lower score of the physical component (OR = 0.27, 95% CI = 0.19–0.39) (Table 7). Women with severe endometriosis were not scoring lower on the SF-12 physical component than women with moderate endometriosis (FDR > 0.1, Supplementary File 1: Table S2). No difference was found in perceived stress, but the major depression inventory (MDI) score was slightly higher in women with endometriosis (OR = 1.74, 95% CI = 1.10–2.65), but they were not more often classified as patients with depression (i.e. MDI score > 20). The SF-12 physical component was not associated with the endometriosis PGS (*P*_FDR_ = 0.66).

**Table 7:** Mental health in women with endometriosis versus controls

Characteristics		*n* (E/C)	Endometriosis	Controls	*P*
SF-12 mental component, mean (SD)		923/22,153	52.3 (7.7)	52.5 (7.6)	1.00
SF-12 physical component, mean (SD)		923/22,153	53.5 (6.5)	55.0 (5.2)	8.36 × 10^−12^
Perceived stress scale, mean (SD)		479/7539	12.3 (5.4)	12.4 (4.9)	1.00
Depression	Classification, %	344/9787	14.0	11.1	1.00
	MDI score, mean (SD)	344/9787	16.3 (4.3)	15.8 (3.9)	0.17

Abbreviations: *n*, number of participants answered the question; *E*, women with endometriosis; *C*, controls; *SF-12*, 12-item short form survey; *MDI*, major depression inventory; *P*_*FDR*_, false discovery rate

### Women with a high genetic risk of endometriosis

Given that endometriosis often remains undiagnosed due to its non-specific symptoms, delays presenting to their doctors, and diagnostic challenges, we investigated whether women with a high genetic predisposition to endometriosis exhibit similar characteristics to those with a confirmed diagnosis. Therefore, we analysed individuals in the highest decile of PGS for endometriosis but without an endometriosis diagnosis, focusing on characteristics that significantly differed between these women and controls. Women with a high genetic risk for endometriosis, but without a diagnosis of endometriosis, more closely resembled controls than women with endometriosis diagnosis across most characteristics (Table 8). However, for two characteristics (postprandial pain and insomnia), women with a high genetic risk of endometriosis resembled women with endometriosis diagnosis, with both symptoms occurring more frequently than in age-matched controls. Women with high PGS but no endometriosis diagnosis were slightly younger than the women with endometriosis diagnosis (44.6 versus 45.7,* P* = 0.01).

**Table 8:** Comparing characteristics of women with high PGS for endometriosis without endometriosis diagnosis, with women with endometriosis diagnosis, and with controls

Trait	*E*	High PGS	*C*	*P*-value	
				**High PGS vs. *E***	**High PGS vs. *C***
Age of onset induced menopause, mean (SD)	40.0 (7.6)	43.3 (7.1)	43.0 (6.7)	< 0.001	0.35
Tried to conceive ≥ 6 months w/o success, %	45.4	23.8	21.1	< 0.001	0.07
Defaecation pattern regularity, irregular, %	18.6	12.7	12.2	0.01	0.67
Abdominal pain, %	29.7	22.4	17.9	0.01	0.004
Postprandial pain, %	14.9	11.3	7.7	0.11	0.001
Defaecation pain, %	15.9	8.6	8.4	< 0.001	0.83
Constipation, %	20.3	15.0	12.3	0.03	0.05
Irritable bowel syndrome, %	20.0	13.2	11.2	0.02	0.17
Insomnia, %	36.7	31.8	28.4	0.14	0.02
Daytime fatigue, %	10.4	6.1	5.3	0.01	0.32
SF12—physical component, mean (SD)	53.5 (6.5)	55.0 (5.3)	54.9 (5.2)	< 0.001	0.46

Abbreviations: *E*, women with endometriosis; *PGS*, polygenic score; *C*, controls

## Discussion

Endometriosis is a heterogeneous, multifactorial disease that presents many different symptoms and comorbidities [9]. In this study, we showed that women with endometriosis have compromised fertility, more gastrointestinal symptoms, and more sleep disturbances compared to age-matched women without endometriosis. We also showed that the PGS of endometriosis was higher in females with endometriosis than in those without endometriosis. While we observed nominally significant associations between the genetic burden of endometriosis and gastrointestinal symptoms, these did not survive correction for multiple testing and should therefore be interpreted with caution. Nonetheless, they may suggest a potential shared genetic component between endometriosis and these comorbidities. However, in individuals without an endometriosis diagnosis, but with a high genetic burden for endometriosis (top decile PGS), we did not observe the same pattern of comorbidities as we saw in women with an endometriosis diagnosis.

### Polygenic score predicting endometriosis

Previously, the polygenic score of endometriosis has been studied to investigate endometriosis [41, 42], and recently, investigate its association with comorbidities [43, 44]. Kloeve-Mogensen et al*.* and Svensson et al*.* used the 14 genome-wide significant loci that were found associated with endometriosis in, at that time, the largest GWAS of endometriosis available [45]. Like the study by McGrath et al., we calculated the PGS based on the most recent GWAS of endometriosis, which increased the number of associated loci [21], and moreover, does not only rely on the genome-wide significant SNPs but include SNPs distributed across the whole genome to capture as much of the genetic variability as possible. Consistent with the previous studies, our results demonstrate a good predictive ability of the endometriosis PGS. Where Svensson et al. did not find any difference between PGS and localization, Kloeve-Mogensen et al. found a better prediction for ovarian endometriosis, though not significantly different than the other localizations. In our study, we confirm the findings of Kloeve-Mogensen et al., and with the increased sample size, we do show a significantly higher polygenic burden of endometriosis among women with severe endometriosis (stage III/IV) compared to those with moderate endometriosis (stage I/II). This finding supports the association between a higher genetic burden and greater disease severity [46, 47]. Furthermore, we confirm this relationship by showing an elevated polygenic burden in individuals with endometriosis affecting multiple locations.

Using the PGS, we investigated whether individuals with a high genetic burden (i.e. top decile PGS, capturing individuals with double risk of endometriosis) but without an endometriosis diagnosis were experiencing similar comorbidities as women diagnosed with endometriosis. While these individuals could provide insight into the consequences of underdiagnosis, in our study they did not show the same patterns of comorbidities as those with a confirmed diagnosis. One explanation is that endometriosis is a highly heterogeneous disease and women with undiagnosed endometriosis might have milder symptoms and did not seek medical attention, or were dismissed when presenting with these symptoms, leading to the perceived lack of a diagnosis and consequently fewer associated comorbidities. Additionally, non-genetic factors, such as hormonal regulation, immune responses, and environmental influences, likely contribute to disease expression and severity, further explaining differences in symptom burden. On the other hand, it is likely that some women in the control population have undiagnosed endometriosis despite not having a high PGS, which could dilute observed associations and bias results toward the null. Another possibility is that the PGS does not fully capture the genetic complexity of endometriosis, as the PGS only moderately distinguishes cases from controls with an AUC of 0.66. While it reflects common genetic variants, endometriosis is a multifactorial disease likely influenced by rare variants, gene–gene and gene-environment interactions, and epigenetic modifications that current PGS models may not adequately account for. Lastly, women with a high PGS but no endometriosis diagnosis were slightly younger than those with a confirmed diagnosis (44.6 vs. 45.7 years; *P* = 0.01). Although this difference was statistically significant, the absolute difference was small and unlikely to fully explain the observed discrepancy in comorbidity patterns. However, we cannot rule out the possibility that some of these women may develop comorbidities later in life, particularly given that participants in the DBDS cohort are relatively young overall (mean age of 44.6 years).

### Endometriosis affects reproductive health

One of the most well-established comorbidities of endometriosis is infertility; lesions can cause structural damage to the reproductive organs, alter hormonal signalling, and create an inflammatory environment that can impair fertility [48, 49]. The elevated rate of women with endometriosis having challenges conceiving in our otherwise healthy cohort confirms the impact of endometriosis on fertility. It is important to note that among participants who responded ‘no’ to ‘tried to conceive ≥ 6 months w/o success’, we were unable to distinguish between those who conceived within 6 months and those who had not attempted to conceive. Further, we found that women with endometriosis reported similar age at menarche compared to controls, while others have reported earlier age at menarche among women with endometriosis [50]. Furthermore, consequent with previous findings [51], we did not find any evidence of longer duration of menstrual bleeding in women with endometriosis, potentially due to the relatively little variation in the data. It has been hypothesized that an earlier, heavier menstruation is a potential cause of endometriosis [52, 53]; we did not find evidence supporting this hypothesis in our cohort. Importantly, the menstrual cycle exhibits substantial inter-individual variation [54], making it particularly susceptible to inaccurate self-reporting and recall bias [55]. However, contraceptive use was not considered in this analysis, potentially affecting results by altering menstrual characteristics such as cycle length and bleeding patterns, thereby obscuring natural associations with endometriosis. Furthermore, use of contraception, and the resulting suppression of menstruation and related symptoms, may also obscure other associations in our study by masking key phenotypic differences. We observed that women with endometriosis experienced induced menopause 3 years earlier than women without endometriosis diagnosis, consistent with a recent large multi-cohort study reporting a 1.6-year earlier onset of surgical menopause [56]. In contrast, we did not observe a significant difference in the timing of natural menopause between women with and without endometriosis.

### Women with endometriosis have more gastrointestinal symptoms

Among women with endometriosis, we observed a higher prevalence of irregular stool pattern and constipation. Moreover, women with endometriosis frequently reported gastrointestinal symptoms, including more abdominal pain, postprandial pain, and defaecation pain. This is in accordance with previous findings showing that women with endometriosis have more bowel symptoms than controls, also when no lesions were present on the bowel [57]. Furthermore, we found more constipation among those with stage III–IV endometriosis compared to those with stage I/II endometriosis; note that endometriosis located at the bowel is categorized under ‘ICD10: N80.8—other endometriosis’ and therefore classified as stage III–IV endometriosis. Further, we found a nominal association between the genetic burden of endometriosis and pain-traits, i.e. abdominal pain and pain during food intake, indicating a potential genetic overlap between endometriosis and those comorbidities.

We found a higher prevalence of self-reported irritable bowel syndrome among women with endometriosis, which is in line with a systematic review that found a threefold increased risk of irritable bowel syndrome in women with endometriosis [58]. Irritable bowel syndrome and endometriosis have overlap in clinical symptoms, such as abdominal discomfort, pain, and cramping. Therefore, it is difficult to distinguish whether the symptoms are caused by endometriosis or irritable bowel syndrome; thus, misdiagnoses might occur as demonstrated by Nnoaham et al. [59]. Furthermore, we found that the endometriosis PGS was nominally significantly associated with self-reported irritable bowel syndrome, suggesting that individuals with a higher genetic predisposition for endometriosis are potentially more likely to report irritable bowel syndrome symptoms. While our data indicate an association, the underlying mechanisms remain to be clarified. One possible explanation is shared genetic architecture between the two conditions, which has been supported by prior studies demonstrating significant genetic correlations, such as the work by Yang et al. [60] Though a study among women with celiac disease showed an increased risk of endometriosis [61], we did not see any increased risk of self-reported celiac disease among women with endometriosis.

### Sleep affected by endometriosis

It has previously been shown that insomnia and fatigue are more prevalent among women with endometriosis [62, 63], which was also confirmed in our study. We did not see an association between the genetic burden of endometriosis and insomnia or fatigue, indicating that the link between endometriosis and sleep is not due to shared genetic factors, but may be a consequence of increased levels of endometriosis-related pain, inflammation, and associated stress as previously demonstrated [64]. Furthermore, it is well known that pain can affect sleep [65]. Whereas we did not find a genetic link between gastrointestinal pain and endometriosis, we did not investigate menstrual-related pain, and the latter may explain the link between endometriosis, pain, and sleep. A recently found association between restless legs syndrome and endometriosis was not confirmed in our study [66].

The complexity of endometriosis and its comorbidities can have a major impact on mental health and overall quality of life, with higher levels of depressive and anxiety symptoms [67, 68]. We identified a non-significant elevated depression score in women with endometriosis compared to the control group, and an assessment using the SF-12 did not indicate a significant impact of endometriosis on mental health, as also demonstrated by Nnoaham et al. [59]. Importantly, the absence of a robust association between endometriosis and mental health in our study could be attributed to the overall good health of our cohort (healthy donor effect) and the young profile, in contrast to a population-based cohort. Moreover, it has previously been shown that women with endometriosis without pelvic pain show no impact on psychological health compared to healthy women [69]. Chronic pelvic pain was not specifically assessed in this study, limiting our ability to evaluate its contribution to psychological health outcomes.

### Strengths and limitations

The DBDS comprises a mainly healthy cohort, as it is the responsibility of any blood bank to ensure that blood donors are healthy at the time of donating blood. For example, individuals on long-term medical treatment are restricted from donating blood, although the use of contraception or paracetamol are not exclusion criteria. This practice introduces a selection bias, known as the healthy donor effect, which has also been shown in the DBDS cohort [70]. As a result, there is an underrepresentation of physical health issues, together with elevated mental health levels, compared to the general population. Indeed, we observed a prevalence of endometriosis of 1.28%, which is significantly lower than the Danish prevalence of endometriosis diagnosed at hospital of 1.63% in 2017 [39]. This suggests that women with endometriosis who are, for example, on long-term medications for the severity of their symptoms, are not blood donors. Importantly, many of our findings are consistent with previous studies, showing that endometriosis does not only affect women experiencing severe symptoms, and reminds us that endometriosis classification and staging does not necessarily correlate with symptoms, treatment response, or prognosis [71]. We confirm this in our study: only constipation was more prevalent among women with stage III/IV endometriosis than among women with stage I/II endometriosis. Furthermore, the actual estimated prevalence of endometriosis in the general population is 10% among women in reproductive age [9]. This discrepancy prompts consideration of potential underdiagnosis, a well-known challenge in endometriosis, which would likely bias associations in our study toward the null resulting in conservative estimates.

In this study, results are based on self-reported questionnaires, where participants’ responses may be subjective and influenced by their personal perspectives, potentially affecting the precision of the data collected. However, high genetic correlations have been found between self-reported diseases and clinically diagnosed diseases [72]. Furthermore, the responses are limited by potential recall bias, influencing the accuracy of participants’ recollection of past events or experiences. In the case of self-reported diseases, and in the case of diseases with overlapping symptoms such as endometriosis and irritable bowel syndrome, we acknowledge the potential for misclassification bias. The potential for confounding variables, sampling bias and the challenge of establishing a clear temporal relationship further contribute to the complexity of interpreting our results.

This study is a retrospective study, and we defined the participant as ‘a case’ when the questionnaire was filled in after a diagnosis of endometriosis. As there is a substantial delay [14–16] in diagnosing endometriosis, this may affect our results in different ways. Firstly, the delayed diagnosis may skew the representation of endometriosis severity, potentially underrepresenting milder cases where individuals have adapted to the disease over time, which affects the generalizability of our results across the entire spectrum of endometriosis severity. Secondly, delayed diagnosis affects the temporal aspect of comorbidity associations. Prolonged exposure to endometriosis without early intervention may contribute to the development of certain comorbidities [18, 59, 73]. Therefore, our findings not only highlight the inherent association between endometriosis and comorbidities but also underscore the impact of delayed diagnosis on these observed relationships. Lastly, both the delay between symptom onset and diagnosis, and the subsequent delay in initiating treatment, may act as confounding factors by influencing the severity or progression of comorbidities. For instance, longer periods without appropriate management may allow comorbid conditions to develop or worsen, thereby exaggerating their association with endometriosis in our data. Variations in treatment regimens and their effectiveness over the extended life course of the disease may influence the prevalence and severity of associated comorbidities in the endometriosis group, necessitating a cautious interpretation of these relationships. Unfortunately, our sample size did not provide enough statistical power to investigate the effect of treatment after receiving a diagnosis.

## Conclusions

Endometriosis is a heterogeneous, multifactorial disease characterized by diverse symptoms and comorbidities. In this study, we demonstrated that women with endometriosis experience compromised fertility, increased gastrointestinal symptoms, and more frequent sleep disturbances compared to age-matched female controls. We also found that the PGS for endometriosis was higher in women with a diagnosis than in those without, and that this genetic burden was nominally associated with gastrointestinal symptoms, indicating a potential shared genetic basis that warrants further investigation. However, women without a diagnosis but with a high genetic predisposition for endometriosis did not exhibit the same broad pattern of comorbidities as seen in diagnosed cases. Our study contributes valuable insights, as well as confirmation of results from previous studies in different cohorts, into the complex interplay between endometriosis and comorbidities, underscoring the need for studying and treating endometriosis as the heterogenous disease that it is.

## Supplementary Information

Supplementary Material 1

## Data Availability

The genetic data that support the findings of this study 28 are available from the DBDS, but re-strictions apply to the availability of these data, which were used under license for the current study, and so are not publicly available. Data are however available with permission of the DBDS steering committee and the national scientific ethical committee. Potential collaborators are encouraged to find additional information and contact details on our homepage: [https://bloddonor.dk/bloddonorstudiet/the-danish-blood-donor-study-eng/](https:/bloddonor.dk/bloddonorstudiet/the-danish-blood-donor-study-eng). Summary statistics from the endometriosis meta-analysis excluding 23andMe are available from the EBI GWAS Catalog (Study Accession GCST90205183). Please note that the Danish cohort was excluded from the generation of summary statistics for this study. Summary statistics from the endometriosis meta-analysis excluding 23andMe are available from the EBI GWAS Catalog (Study Accession GCST90205183). Please note that the Danish cohort was excluded from the generation of summary statistics for this study.

## Abbreviations

DBDS: Danish Blood Donor Study
PGS: Polygenic score
OR: Odds ratio
GWAS: Genome-wide association studies
SF12: Short Form Health Survey 12
DNPR: Danish National Patient Registry
PCs: Principal components
LD: Linkage disequilibrium
PCS: Physical component summary score
MCS: Mental component summary score
PSS: Cohen’s Perceived Stress Scale
MDI: Major Depression Inventory
AUC: Area under the curve
FDR: False discovery rate
SD: Standard deviation
